# Association Between Circular RNAs and Intracranial Aneurysm Rupture Under the Synergistic Effect of Individual Environmental Factors

**DOI:** 10.3389/fneur.2021.594835

**Published:** 2021-03-04

**Authors:** Qing Huang, Yi Sun, Qiuyu Huang, Yile Zeng, Shaowei Lin, Shuna Huang, Yingying Cai, Xingyan Xu, Dezhi Kang, Huangyuan Li, Siying Wu

**Affiliations:** ^1^Department of Neurosurgery, The Second Affiliated Hospital of Fujian Medical University, Quanzhou, China; ^2^School of Public Health, Fujian Medical University, Fuzhou, China; ^3^Department of Neurosurgery, The First Affiliated Hospital of Fujian Medical University, Fuzhou, China

**Keywords:** individual environmental factors, circular RNAs, intracranial aneurysm, high-throughput sequencing, diagnostic value

## Abstract

**Introduction:** To study the association between specific circular RNAs and rupture of intracranial aneurysm. To explore its clinical diagnostic significance and synergistic effects with individual environmental influencing factors.

**Methods:** Three hundred and forty seven cases and controls were included in this study. Multivariate analysis was used to explore the main individual environmental factors. Intracranial aneurysm rupture related circular RNAs screened based on sequencing was verified in peripheral blood by PCR. ROC curve, logistic regression model and fork analysis were used to study the association, diagnostic values, and synergistic effects of circular RNA with intracranial aneurysms and individual environmental factors.

**Results:** Smoking, hair dyeing, sitting time ≥6 h/day, single animal oil intake and hypertension are the main risk factors for intracranial aneurysm rupture; People with higher education, sleeping time ≥7 h/day, tea drinking, diabetes, higher levels of (hemoglobin, low density lipoprotein, serum calcium, and apolipoprotein-A1) have a low risk of intracranial aneurysm rupture. Hsa_circ_0008433 and hsa_circ_0001946 are closely related to intracranial aneurysm rupture and have certain clinical diagnostic significance (AUC = 0.726; 95% CI: 0.668~0.784). Hsa_circ_0008433 (OR = 0.497, 95% CI: 0.338~0.731), hsa_circ_0001946 (OR = 0.682, 95% CI: 0.509~0.914) were independent epigenetic factors affecting intracranial aneurysm rupture, and have a multiplicative interaction with age (OR = 3.052, 95% CI: 1.006~9.258).

**Conclusions:** Low expressions of hsa_circ_0008433 and hsa_circ_0001946 are risk factors for intracranial aneurysms rupture and have good clinical diagnostic value. There was a multiplicative interaction between epigenetic score and age. The older and the higher the epigenetic score was, the more likely to have intracranial aneurysm rupture.

## Introduction

Intracranial aneurysm (IA) is a localized abnormal bulging caused by high blood flow due to congenital weakness of the cerebral artery wall or acquired lesions (1). About 0.7 to 1.9% of IA can rupture under various inducements, leading to aneurysmal subarachnoid hemorrhage. The annual incidence of IA rupture worldwide is about 9.1 per 100,000, accounting for 3 to 5% of all acute strokes. The age of onset is between 40 and 60 years old, the mortality rate is close to 40%, and 46% of survivors can be disabled or long-term cognitive impairment due to multiple complications (2–5). Therefore, it is called the “time bomb” in the brain, which seriously threatens human life and health (6). Current research showed that the traditional risk factors for IA rupture are smoking, alcoholism, high blood pressure, gender, drugs, and blood lipid levels, etc (7–13). However, under the current social and economic development, the tremendous changes in people's life and habits will inevitably be accompanied by new individual environmental impact factors, and these new impact factors are not yet very clear.

Circular RNA is a type of novel nucleic acid molecule that does not have a characteristic 5'cap end and 3'poly (A) end, and exists in the form of a covalent loop (14). It is widely distributed, highly conservative in structure, and has specificity of temporal expression and spatial distribution. At present, circRNA has rapidly become a hot spot in the field of cerebrovascular disease research, and has been deeply explored as an emerging biological marker and therapeutic target (15). Recent studies have found that circRNA can competitively bind to specific miRNA through sponge adsorption to regulate the expression of downstream target genes. The related mechanism is called competitive endogenous RNA (ceRNA). A large number of subsequent studies have confirmed that the ceRNA mechanism involves many important pathological processes such as inflammation, SMC phenotype transformation and extracellular matrix, which play an important role in epigenetic regulation in vascular diseases (16, 17). However, there are still few studies on the relationship between circRNA and IA rupture recently and its synergistic effects with individual environmental factors are even rarer.

In this study, a questionnaire survey was conducted on 347 cases of IA rupture and 347 healthy examinees. Logistic regression analysis was used to study the individual environmental factors that affect the rupture of IAs. At the same time, based on the previous high-throughput sequencing results, the selected IA rupture-related circRNA were verified in the population peripheral blood, the potential correlation of these specific circRNA expression patterns in IA tissues and peripheral blood was further explored.

## Materials

### Study Population

A total of 347 patients with IA rupture and 347 healthy persons who were admitted to the Neurosurgery and Physical Examination Center of the Second Affiliated Hospital of Fujian Medical University from June 2017 to September 2019 were selected as case and control groups for individual environmental factors study. At the same time, 140 subjects with matching baseline data were selected from the case and the control group, their peripheral blood was collected for PCR experiments. Case group inclusion criteria: ① Age> 18 years old, and first onset; ② IA rupture confirmed by computed tomography angiography (CTA), magnetic resonance angiography (MRA), or digital subtraction angiography (DSA); ③ There was no significant systemic disease that might affect the indexes; The medical record is complete and willing to cooperate with the questionnaire. Case group exclusion criteria: ① Those with the first head CT scan or negative lumbar puncture; ② Patients with a clear history of subarachnoid hemorrhage or a family history of severe cerebrovascular disease; ③ Those with previous stroke, brain tumor, or history of craniocerebral surgery; ④ Patients with coagulopathy, fever, or pregnancy. Control group inclusion criteria: ① Immediate relatives of IA or a family history of a clear subarachnoid hemorrhage; ② Patients with previous stroke, brain tumor, or history of craniocerebral surgery; ③ Recently infected or pregnant; ④ Patients with systemic severe chronic diseases; ⑤ People with incomplete clinical data or refuse to participate in this study. All research subjects signed an informed consent form and approved by the Ethics Committee of the Second Affiliated Hospital of Fujian Medical University (2018-50).

### Questionnaire

A unified structured questionnaire is adopted, and the research objects are investigated by strictly trained staff with certain epidemiological and clinical work experience. The content includes general conditions such as gender, age, occupation, education, marital status, personal medical history, and so on. Individual behaviors include smoking, drinking alcohol and tea, chemical poison exposure, taking medicine, sitting and sleeping time per day, physical exercise and labor intensity. Eating habits mainly include eating habits and edible oil types. Define smokers who smoke ≥1 cigarette per day for >6 consecutive months or cumulatively smoke ≥100 cigarettes; Define a single drinking volume of ≥50 g as drinking; Drinking tea ≥1 cup per week for more than 6 months is defined as drinking tea; Actively participate in sports activities at least once a week and last at least 20 min each time is defined as physical exercise (18). The daily sleep time of adults over 18 years old <7 h is defined as insufficient sleep, and the daily sleep time ≥9 h is defined as excessive sleep (19). Sedentary or semi-recumbent time >6 h was definited as sedentary (20).

### RNA Extraction

Two milliliters peripheral venous blood was extracted and centrifuged. Lymphocyte separation solution was added to the blood cell layer, the suspension layer of white blood cells was further extracted. Then absorb the leukocyte suspension layer and add Trizol to fully lyse, further extract the total RNA of the peripheral blood according to the kit instructions. The NanoDrop® ND-2000 nucleic acid quantifier was used to determine the RNA content. The specimens with OD260/280 values between 1.80 and 2.00 were considered qualified samples.

### cDNA Synthesis and PCR

Strictly follow the instructions of PrimeScript^TM^ RT reagent Kit-RR037 kit for cDNA synthesis of circular RNA. According to the instructions of the TB Green^TM^ Premix EX Taq^TM^-RR820 kit, the Real-Time PCR System amplifier (Light Cycler 480) was used for qRT-PCR detection, establish 20 μl system. The reaction conditions are: Pre-denaturation 95°C (30 s), 1 cycle; PCR reaction 95°C (5 s), 60°C (34 s), 45 cycles; melting reaction 95°C (15 s), 60°C (1 min), 95°C (15 s), 1 cycle. Take GAPDH as internal reference.

### Statistical Analysis

Two independent sample *t*-tests were used to compare the difference between normal distribution quantitative data. Two independent sample rank sum tests (Mann-Whitney *U*) were used to analyze quantitative data that did not obey the normal distribution. Chi-square test was used to infer the overall rate or composition ratio. Multivariate logistic regression was used to analyze the odds ratio (OR) of each variable and the risk of IA rupture and its 95% confidence interval (95% CI). Real-time fluorescence quantitative PCR results are expressed by -ΔΔCT method. To study the interaction between epigenetic indicators and environmental factors by using fork analysis, and further calculate the interaction index (S), attribution ratio (AP), and excess relative risk (RERI).

## Result

### General Demographic Characteristics

A total of 347 cases and controls were included using the propensity score method. Among them, 392 were male (56.48%) and 302 were female (43.51%), with an average age of 51.91 ± 10.15 years. The general demographic characteristics of the case group and the control group are balanced and comparable (*p* > 0.05) (Supplementary Table 1).

### Univariate Analysis of Individual Environmental Factors in IA Rupture

#### Correlation Between Behavioral Factors and IA Rupture

The analysis of behavioral factors showed that there were statistical differences between the case and control group in education level, exposure to chemical poisons, daily sitting and sleeping time, physical exercise, tea drinking, and smoking (*p* < 0.05) (Supplementary Table 2).

#### Correlation Between Eating Habits and IA Rupture

The analysis of the eating habits showed that the salty and light diet, edible oil types were statistically different between the case and the control group (*p* < 0.05) (Supplementary Table 3).

#### Correlation Between Specific Physiological Indicators and IA Rupture

The analysis of the physiological indicators showed that diastolic blood pressure and the pulse pressure were statistically different between the case and the control group (*p* < 0.05) (Supplementary Table 4).

#### Correlation Between Specific Biochemical Indicators and IA Rupture

The analysis of the biochemical indicators showed that hemoglobin, low density lipoprotein, triglyceride, cholesterol, serum calcium, apolipoprotein A1, and apolipoprotein B were statistically different between the case and the control group (*p* < 0.05) (Supplementary Table 5).

#### Correlation Between Disease History and IA Rupture

The analysis of the biochemical indicators showed that hypertension and stroke disease and family history are statistically different between the case group and the control group (*p* < 0.05) (Supplementary Table 6).

### Logistic Regression Analysis of Individual Factors of IA Rupture

Taking IA rupture as the dependent variable, the single factors with statistically significant difference were further included in logistic regression model for analysis (Backward: Wald). The results showed that smoking, chemical poison exposure (hair dye), sitting time >6 h/day, single animal oil intake, hypertension, larger diastolic pressure and pulse pressure differences, higher levels of plasma globulin are the risk factors for IA rupture. Tea drinking, higher education, sleep time >7 h/day, diabetes, and higher levels of (hemoglobin, low density lipoprotein, apolipoprotein A1, and serum calcium) are the protective factors for IA rupture (Table 1).

**Table 1:** Logistic regression analysis of individual factors of IA rupture.

**Variable**	**β**	**S.E**	**Wald χ^2^**	***p***	**OR (95% CI)**
Education level			16.585	<0.001	
Primary school					
Middle school	−0.208	0.232	0.804	0.370	0.812 (0.515~1.280)
University	−1.125	0.290	15.035	<0.001	0.325 (0.184~0.573)
Poison exposure No Organic reagents Pesticides Chemical	0.558 −0.949 −0.434	0.256 0.685 0.584	7.698 4.757 1.920 0.554	0.053 0.029 0.166 0.457	1.747 (1.058~2.883) 0.387 (0.101~1.482) 0.648 (0.206~2.033)
Sitting time	1.218	0.503	5.868	0.015	3.382 (1.262~9.062)
Sleep time	−0.718	0.352	4.170	0.041	0.488 (0.245~0.972)
Tea drinking No 1~4 /week ≥5 /week	−0.644 −0.899	0.231 0.313	11.261 7.746 8.273	0.004 0.005 0.004	0.525 (0.334~0.827) 0.407 (0.221~0.751)
Somking No Quit smoking Smoking now	−0.245 0.934	0.416 0.252	16.782 0.347 13.717	<0.001 0.556 <0.001	0.783 (0.346~1.769) 2.545 (1.552~4.172)
Drinking			6.333	0.096	
No					
1~2 /week	−0.910	0.480	3.589	0.058	0.403 (0.157~1.032)
3~4 /week	−2.099	1.238	2.874	0.090	0.123 (0.011~1.388)
≥5 /week	−0.217	0.563	0.149	0.699	0.805 (0.267~2.424)
Salty diet	0.428	0.240	3.165	0.075	1.534 (0.957~2.456)
Edible oil type			4.816	0.090	
Vegetable oil					
Animal oil	0.970	0.468	4.295	0.038	2.638 (1.054~6.602)
Mixed food	0.278	0.267	1.083	0.298	1.321 (0.782~2.231)
Diastolic pressure	0.045	0.009	26.789	<0.001	1.046 (1.028~1.064)
Pulse pressure	0.020	0.006	9.413	0.002	1.020 (1.007~1.033)
Hb	−0.017	0.007	6.456	0.011	0.983 (0.971~0.996)
GLB	0.072	0.024	8.884	0.003	1.075 (1.025~1.127)
LDL	−0.221	0.104	4.510	0.034	0.802 (0.654~0.983)
Ca^2+^	−1.907	0.657	8.425	0.004	0.149 (0.041~0.538)
Apo-A1	−0.752	0.305	6.082	0.014	0.471 (0.259~0.857)
Hypertension	0.857	0.227	14.257	<0.001	2.355 (1.510~3.675)
Diabetes	−1.303	0.538	5.865	0.015	0.272 (0.095~0.780)

### Expression of IA Rupture-Related circRNAs in Peripheral Blood

**Figure 1:**
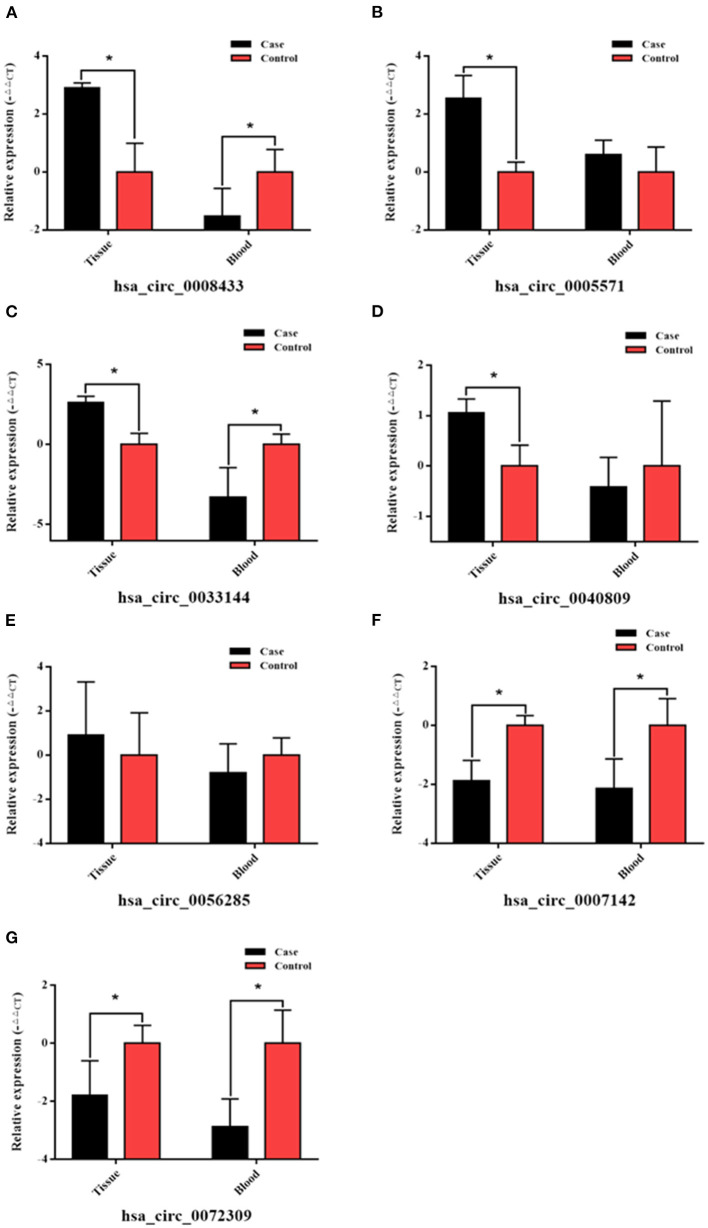
qRT-PCR verification of specific circRNAs in IA tissue and peripheral blood of case and control group. **(A–G)** The PCR results of the 7 selected circRNAs hsa_circ_ (0008433, 0005571, 0033144, 0040809, 0056285, 0007142, 0072309) in IA tissue and peripheral blood, *P < 0.05.

Based on the previous high-throughput sequencing results, seven IA rupture-related circRNAs were selected (Supplementary Table 7), and the reverse splicing technology was used in circ RNA primer design (Table 2). Their expression in the peripheral blood of the same patient (the one who provided tissue samples for RNA-Seq) was verified (*n* = 4). The results showed that hsa_circ_0008433 and hsa_circ_0005571 were both highly expressed in IA tissues (*p* < 0.05), but the expression of them in peripheral blood was low and high, respectively (Figures 1A,B). Similar to hsa_circ_0008433, hsa_circ_0033144 was highly expressed in IA tissues and low in peripheral blood of the two groups (Figure 1C, *p* < 0.05). Hsa_circ_0040809 was highly expressed in IA tissues and low in peripheral blood, but the differences were only statistically significant in the tissue expression (Figure 1D, *p* < 0.05). Hsa_circ_0056285 was highly expressed in IA tissues and low in peripheral blood, but the differences were not statistically significant (Figure 1E, *p* > 0.05). Hsa_circ_0007142 and hsa_circ_0072309 showed low expression both in IA tissues and peripheral blood, the differences between cases and control group of the two samples were statistically significant (Figures 1F,G, *p* < 0.05).

**Table 2:** CircRNA and internal reference gene primer sequences.

**circRNA**	**Primer**	**Sequence (5^**′**^-3^**′**^)**
β-actin (H)	Forward	5′ GTGGCCGAGGACTTTGATTG 3'
	Reverse	5′ CCTGTAACAACGCATCTCATATT 3′
hsa_circ_0008433	Forward	5′ TCCAAGCATTGCTATTACAACTG 3′
	Reverse	5′CCCTCTTAGGATGTCTGTTATTCA 3′
hsa_circ_0033144	Forward	5′ TGCTCTCACCCACGAAAGG 3′
	Reverse	5′ CTCCACATGGTCAGCCTCTG 3′
hsa_circ_0005571	Forward	5′ TGCGTGTTGGATGAACTTGA 3′
	Reverse	5′ AGGTATAGATTGCCTGTTAGTGG 3′
hsa_circ_0040809	Forward	5′ GCAACAAAGTGCGATGGTGA 3′
	Reverse	5′ CAGCTCTGTACCTGGGTGGTC 3′
hsa_circ_0007142	Forward	5′ TCACAAATTCTTTCTGGAACTCTG 3′
	Reverse	5′ CCGCTCCTCTGGCATCATA 3′
hsa_circ_0072309	Forward	5′ AGTTTTTCCACACCGCTCAA 3′
	Reverse	5′ TCCAGGATGGTCGTTTCAA 3′
hsa_circ_0056285	Forward	5′ GCGTGCAGTACGTGGAGAC 3′
	Reverse	5′ GTCTTCTACAAACTCGTCATACATG 3′
hsa_circ_0001946	Forward	5′ AGTCTTCCATCAACTGGCTCA 3′
	Reverse	5′ GACACAGGTGCCATCGGA 3′
hsa_circ_0000284	Forward	5′ TATGTTGGTGGATCCTGTTCGGCA 3′
	Reverse	5′ TGGTGGGTAGACCAAGACTTGTGA 3′

**Figure 2:**
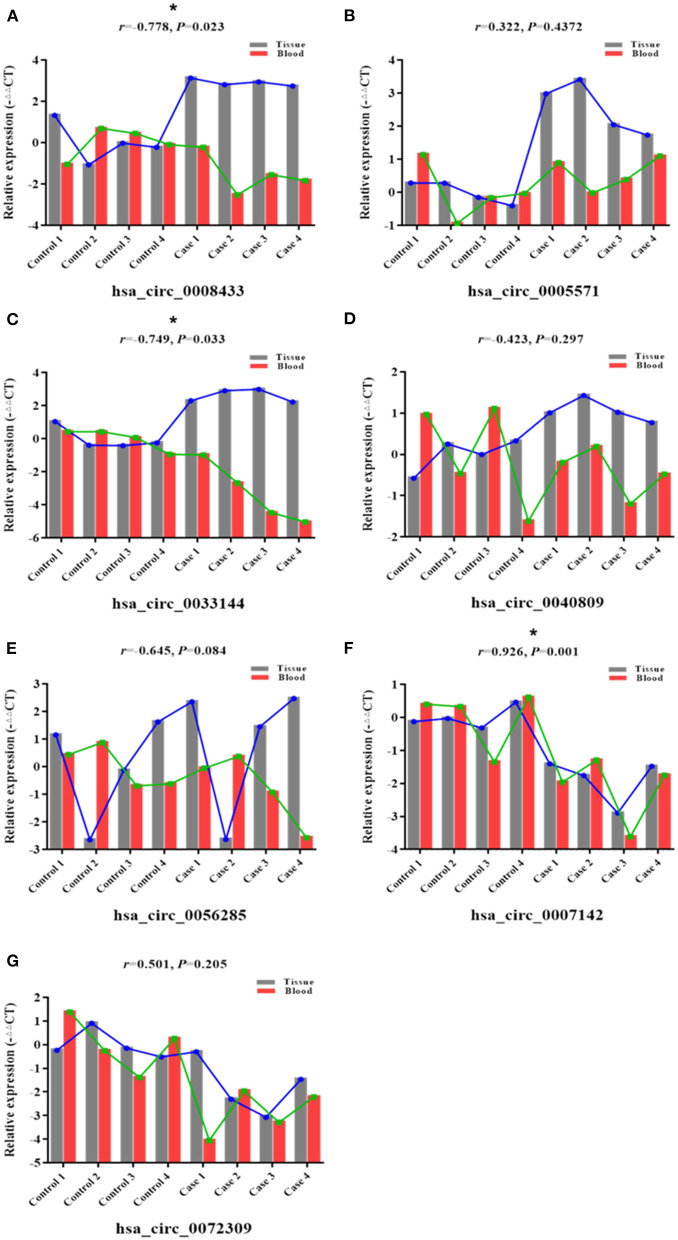
Correlation between specific circRNAs in IA tissue and blood. **(A–G)** Correlations between 7 selected circRNAs hsa_circ_ (0008433, 0005571, 0033144, 0040809, 0056285, 0007142, 0072309) in each IA and STA sample from tissue and peripheral blood, *P < 0.05.

To further study the potential association of circRNA expression in tissue and peripheral blood, the expression of the above-mentioned specific IA rupture-related circRNAs in the same patient's IA tissues and peripheral blood was described (Figures 2A–G). Pearson correlation analysis was used to study the expression patterns and correlations of these specific circRNAs in two samples of the same patient. The results showed that the expression of hsa_circ_0008433 (*r* = −0.778, 95% CI: −0.958 to −0.163) and hsa_circ_0033144 (*r* = −0.749, 95% CI: −0.951 to −0.093) in IA tissues was negatively correlated with that in peripheral blood (Figures 2A,C). The expression of hsa_circ_0007142 (*r* = 0.926, 95% CI: 0.635~0.987) in IA tissues was positively correlated with that in peripheral blood (Figure 2F).

### Detection of Peripheral Blood PCR in Small Sample Population

#### Screening circRNA Indicators for PCR Detection

Based on the RNA-Seq results and combined with the preliminary experiments (21), we further selected 4 of the 7 circRNAs verified above for PCR detection in the population peripheral blood. Among them, the expression differences of hsa_circ_0005571, hsa_circ_0033144, and hsa_circ_0007142 in case and control group of IA tissues and peripheral blood were statistically significant (*p* < 0.05); hsa_circ_0008433 had a large differential expression multiple in RNA-Seq (FC = 52.077). At the same time, hsa_circ_0001946 (circ-CDR1as) and hsa_circ_0000284 (circ-HIPK3) were selected by reviewing the literature, they are two circRNAs closely related to cerebrovascular diseases that are currently being noticed (Table 3) (22, 23).

**Table 3:** CircRNAs for PCR in peripheral blood of small sample population.

**circRNA ID**	**Host gene**	**Length (nt)**	**Log2 FC**	***p***
hsa_circ_0008433	PDE4B	351	5.7026	<0.001
hsa_circ_0033144	BCL11B	369	3.8851	<0.001
hsa_circ_0005571	IFI30	658	3.6338	<0.001
hsa_circ_0007142	DOCK1	427	−4.2062	0.003
hsa_circ_0001946	CDR1	1,485		
hsa_circ_0000284	HIPK3	1,099		

#### qRT-PCR Detection of circRNA

**Figure 3:**
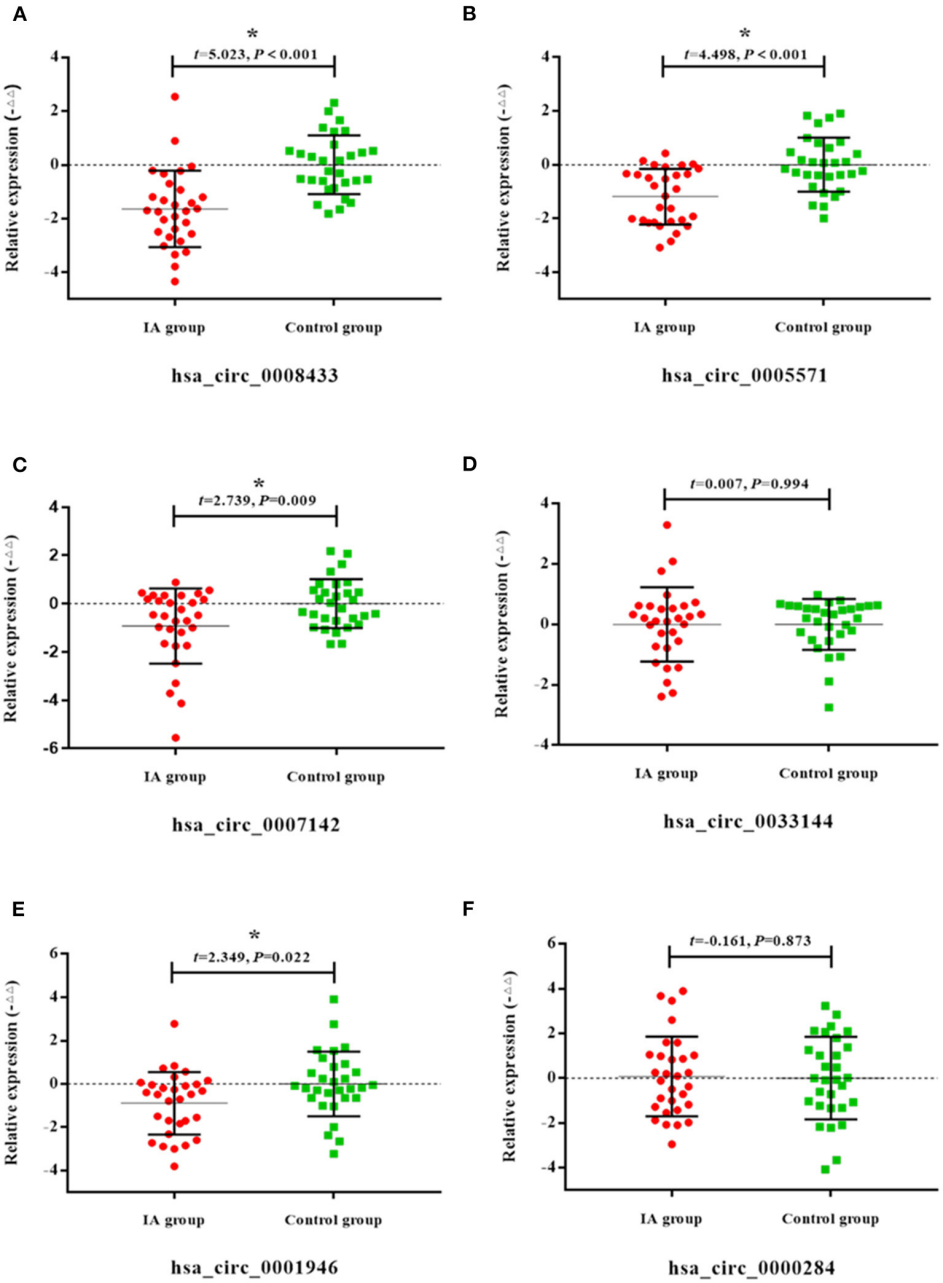
Expression of specific circRNAs in peripheral blood. **(A–F)** Population qRT-PCR verification of hsa_circ_ (0008433, 0005571, 0007142, 0033144, 0001946, 0000284) expression in peripheral blood of IA and control group, IA group (*n* = 30) vs. control group (*n* = 30), *P < 0.05.

Peripheral blood of 30 patients with IA rupture and 30 healthy people were collected for qRt-PCR. The results showed that the expressions of hsa_circ_0008433, hsa_circ_0005571, hsa_circ_0007142, hsa_circ_0001946 in peripheral blood of patients with IA rupture were lower than that of the control group, and the difference was statistically significant (*p* < 0.05) (Figure 3).

### Detection of Peripheral Blood PCR in Expanded Sample Population

#### Study Population and General Demographic Characteristics

The peripheral blood of 140 patients with IA rupture and 140 healthy people were further collected for PCR verification in expanded sample population. The general demographic characteristics of the case and the control group were balanced and comparable (*p* > 0.05) (Supplementary Table 8).

#### qRT-PCR Detection of circRNA

**Figure 4:**
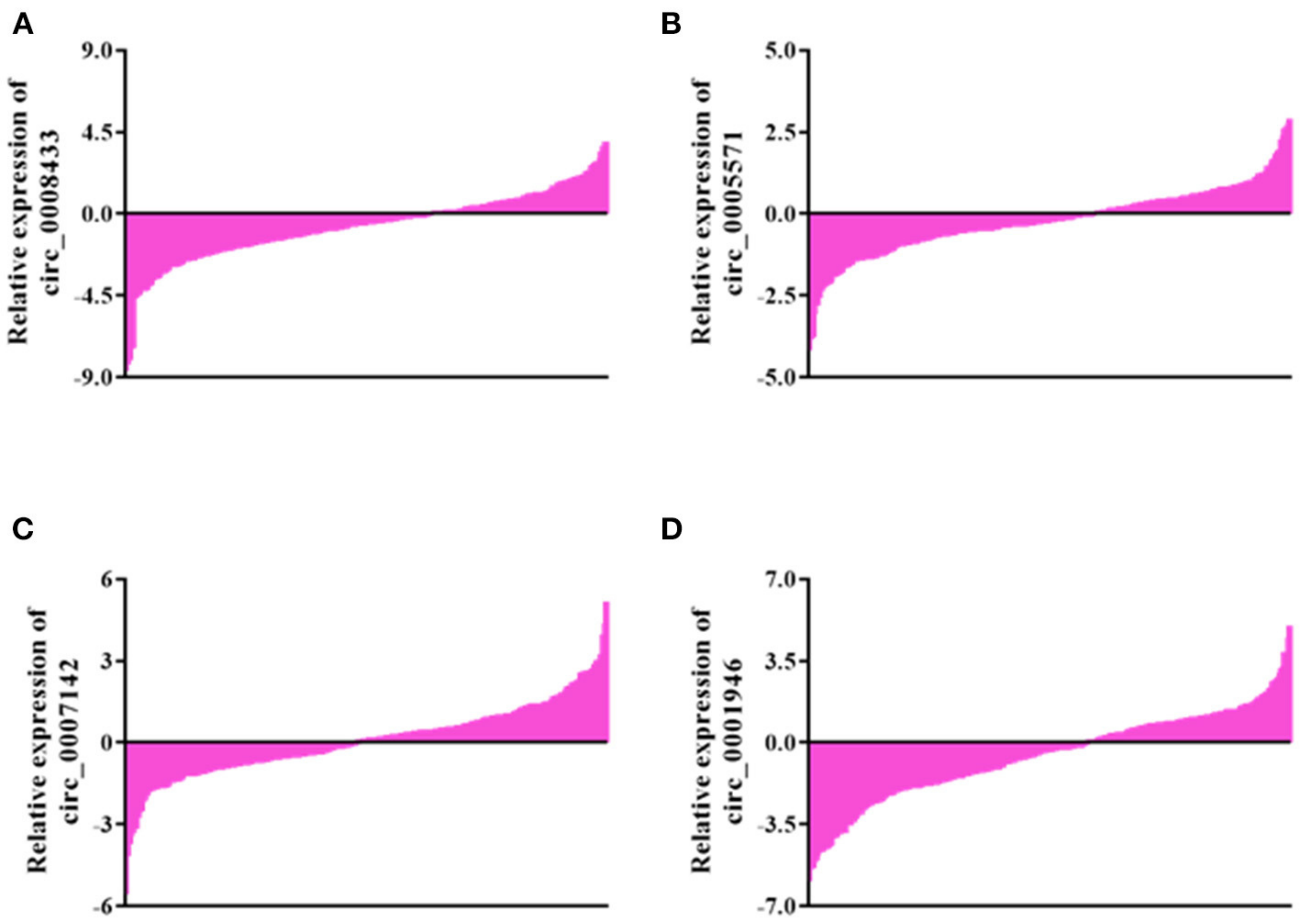
General expression of specific circRNAs in blood of population. **(A–D)** General expression of hsa_circ_ (0008433, 0005571, 0007142, 0001946) in blood of population.

Select 4 IA rupture-related circRNA indicators (hsa_circ_0008433, hsa_circ_0005571, hsa_circ_0007142, hsa_circ_0007142, hsa_circ_0001946) with significant expression differences in small sample population peripheral blood for the next verification in an expanded sample population. The results showed that compared with the control group, 75.0% (105/140), 65.71% (92/140), 55.71% (78/140), and 67.14% (94/140) of patients showed down-regulation of the four circRNAs in peripheral blood (Figure 4).

**Figure 5:**
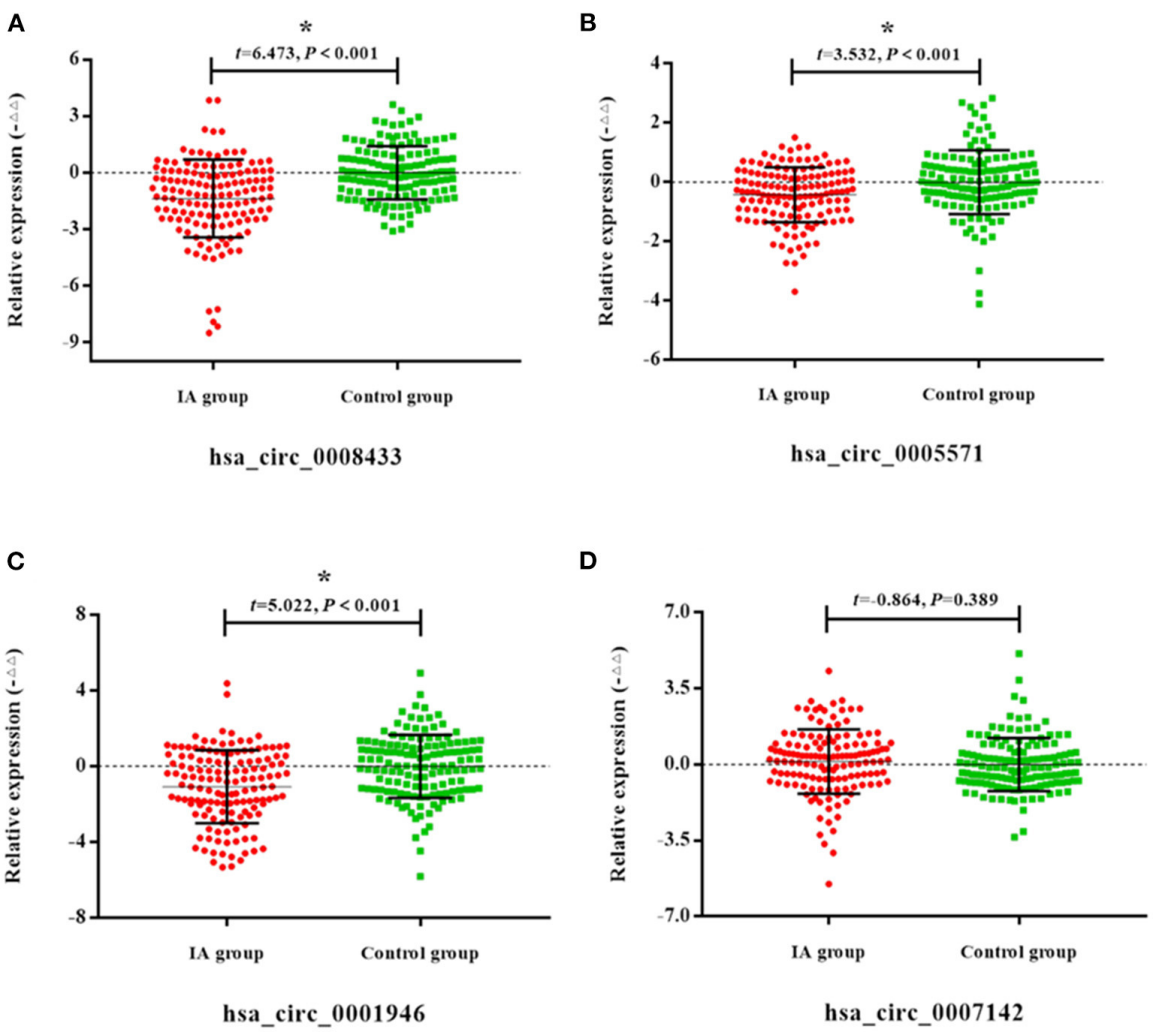
Expression of specific circRNAs in peripheral blood. **(A–D)** Population qRT-PCR verification of hsa_circ_ (0008433, 0005571, 0001946, 0007142) expression in peripheral blood of IA and control group, IA group (*n* = 140) vs. control group (*n* = 140), *P < 0.05.

The results of qRT-PCR showed that compared with the control group, the expression of hsa_circ_0008433, hsa_circ_0005571, and hsa_circ_0001946 in the peripheral blood of patients with IA were down-regulated, and the difference was statistically significant (Figures 5A–D, *p* < 0.05).

### Association of circRNA and IA Rupture

A logistic regression model was established to analyze whether these specific circRNAs were independent influencing factors for IA rupture. The results showed that after adjustment of the main individual environmental factors, has_circ_0008433 (OR = 0.497, 95% CI: 0.338~0.731) and has_circ_0001946 (OR = 0.682, 95% CI: 0.509~0.914) were independent influencing factors of IA rupture. High-level expression of has_circ_0008433 and has_circ_0001946 in peripheral blood may have a protective effect on IA rupture (Table 4).

**Table 4:** Multivariate analysis of circRNA and IA rupture.

**circRNA**	**OR**	**95% CI**	**^a^OR**	**^a^95% CI**
has_circ_0008433	0.613	0.516~0.727	0.497	0.338~0.731
has_circ_0005571	0.648	0.504~0.835	0.857	0.535~1.374
has_circ_0001946	0.717	0.622~0.827	0.682	0.509~0.914

a *Is the adjustment of sitting and sleeping time, drinking tea, smoking, edible oil, diastolic blood pressure, pulse difference, hemoglobin, globulin, LDL, serum calcium, Apo-A1, hypertension, and diabetes adjustment*.

### ROC Curve

**Figure 6:**
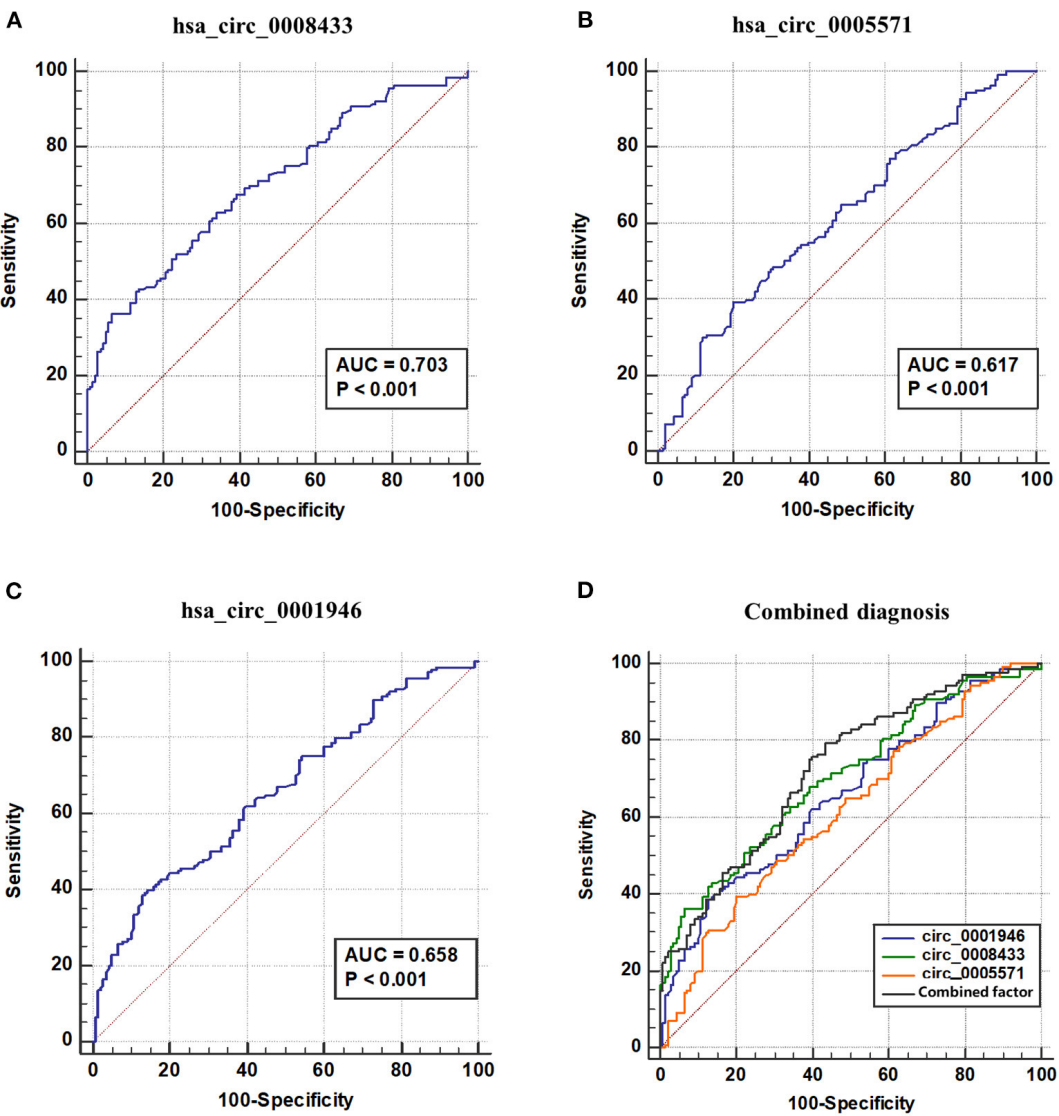
ROC curve of IA related circRNAs and combined diagnosis. **(A–D)** ROC curve of hsa_circ_ (0008433, 0005571, 0001946) and combined factor. AUC, Area under ROC curve.

ROC curve is used to explore the significance of specific circRNA in the diagnosis of IA rupture. The results show that the area under the ROC curve (AUC) of has_circ_0008433 is 0.7028, the maximum Youden index is 0.3000, and the corresponding cutoff is −1.9051 (Figure 6A); The AUC of has_circ_0005571 is 0.617, the maximum Youden index is 0.1928, and the corresponding cutoff is −0.5889 (Figure 6B); The AUC of has_circ_0001946 is 0.664, the maximum Youden index is 0.2571, and the corresponding cutoff is −1.6451 (Figure 6C, Supplementary Table 9).

The three circRNAs were further adopted into the logistic regression equation to construct new combined diagnostic factors and draw ROC curves. The results showed that the AUC of the new combined diagnostic factor was 0.726 (95% CI: 0.668~0.784), the sensitivity and specificity were 0.793 and 0.564, respectively. Further comparison test results of the ROC curves of these circRNA and new combined factors showed that, hsa_circ_0008433 has higher diagnostic recognition than has_circ_0005571, and when the three circRNAs were jointly predicted, the diagnostic value is better than that of circ_0005571 or circ_0001946 single index (*p* < 0.05) (Figure 6, Supplementary Table 10).

### The Combined Effects of Epigenetics and Other Influencing Factors

has_circ_0008433, has_circ_0005571, and has_circ_0001946 were further adopted in logistic regression model, the case and the control group were divided into high and low risk score according to the median epigenetic risk score of the control group, and the epigenetic IA risk score was constructed (the higher the score, the greater the risk of IA rupture), so as to explore the joint effects of circRNA epigenetic risk and age. The results show that patients with high epigenetic risk and older (≥55 years old) have an increased risk of IA rupture (*p* < 0.05), which indicated that there is a certain multiplicative interaction between epigenetic factors and age (Table 5).

**Table 5:** Combined effects of epigenetic risk and age on IA.

**Age**	**Genetic risk**	**Case/Control**	**^a^OR (95% CI)**
<55	Low	23/50	1.000
<55	High	59/45	2.872 (1.497~5.511)
≥55	Low	10/29	0.816 (0.333~2.000)
≥55	High	16/48	7.981 (3.609~17.649)
Age × Genetic point			3.052 (1.006~9.258)
*RERI*			3.501 (-0.167~7.168)
*AP*			1.147 (1.039~1.255)
*S*			−1.418 (−2.314~3.365)

a *Is adjusted by gender, marital status, education level*.

## Discussion

Current studies suggest that IA is a complex disease with multiple environmental factors and genetic regulation (24). This study indicated that people with smoking, chemical poison exposure (hair dye), sitting time >6 h/day, single animal oil intake, larger diastolic pressure and pulse pressure differences, higher levels of plasma globulin have higher risk of IA rupture. On the contrary, people with tea drinking habits, higher education, sleep time >7 h/day, diabetes and higher levels of (hemoglobin, low density lipoprotein, apolipoprotein A1, and serum calcium) have a lower risk of IA rupture. After controlling these major individual environmental factors, the low expression of circular RNA has_circ_0008433 and has_circ_0001946 in peripheral blood is the independent epigenetic risk factors for IA rupture. At the same time, the epigenetic risk score constructed based on these two circular RNAs interacts with age and has a certain clinical diagnostic values for IA repture.

It is worth mentioning that, in addition to the traditionally recognized influencing factors (smoking, drinking, biochemical indicators changes, etc.), this study also has some new findings. For example, people with higher diastolic pressure and pulse pressure differences are susceptible to IA rupture. Our data shows that for every 1 mmHg increase in diastolic blood pressure, the risk of IA rupture increases by about 4.6% (OR = 1.046, 95% CI: 1.007~1.033). For every 1 mmHg increase in pulse pressure difference, the risk of IA rupture increases by about 2.1% (OR = 1.021, 5% CI: 1.007~1.033). Studies have shown that patients with simple diastolic hypertension have a higher incidence of cerebrovascular disease. In Asian and European populations, for every 5 mmHg decrease in diastolic blood pressure, the risk of stroke is reduced by 44 and 27%, respectively (25). At the same time, the increase in pulse pressure difference means that the stress load on the blood vessel wall increases, which will lead to changes in arterial structure and decreased compliance, that constitutes a local weakening factor of the blood vessel wall (26, 27). Therefore, more attention should be paid to the monitoring and management of diastolic blood pressure and pulse pressure difference in the control of IA rupture, which may have more positive significance for its prevention.

Sedentary activity has been listed by the American Heart Association (AHA) as one of the independent risk factors for cerebrovascular diseases (28). According to research data, the all-cause mortality rate of people who sit for more than 11 h a day will increase by 40% (29). Sedentary reduces muscle contraction and blood circulation, promotes an increase in arterial pressure. In addition, pathological changes such as oxidative stress and endothelial dysfunction induced by continuous high perfusion become an important pathological basis for the occurrence of vascular diseases (30). In our data, the OR of people with sitting time> 6 h/day suffering from IA rupture was 3.382 (95% CI: 1.262~9.062). Meanwhile, hair dye exposure is probably another bad lifestyle that may induce IA rupture. P-phenylenediamine (PPD) in hair dyes has a variety of target organ toxicity, which can induce TP53 gene mutation and cause DNA damage (31, 32). Although there is no direct report that the hair dye is related to the occurrence of IA, PPD as an environmental chemical poison and a new potential influencing factor of IA still deserve our attention.

Recent studies have shown that inflammation is the most important pathological mechanism in IA rupture, and circRNA may play an epigenetic regulatory role in the pathological mechanism of IA through various inflammatory pathways (33–35). including regulation of inflammatory gene expression, recruitment, chemotactic aggregation of macrophages and regulation of key signaling pathways, etc (36). In this study, based on high-throughput sequencing to analyze the circRNA expression profiles in IA tissues and their biological functions. The results showed that these abnormally expressed IA-related circRNAs are closely related to inflammation, and some important biological processes and cell signal pathways that associate with inflammation may be regulated by circular RNA epigenetic regulation.

Among them, the expressions of hsa_circ_0008433 and hsa_circ_0005571 in IA tissues and normal blood vessels are significantly different, and they still show such expression differences in the subsequent peripheral blood verification to expand the sample size. After adjusting for individual environmental factors that are important for the rupture of IA, the circular RNAs hsa_circ_0008433, hsa_circ_0005571, and hsa_circ_0001946 are independent epigenetic influencing factors for IA rupture, and have a certain synergistic interaction with age (>55 years). The results indicated that circRNA may participate in the pathological process of IA through various of mechanisms, which provide certain references for the molecular etiology of IA rupture (37–40).

At the same time, a “star molecule” of circular RNA circ-CDR1as (hsa_circ_0001946) which is widely studied currently was selected for peripheral blood PCR verification of IA rupture. The results showed that the expression of hsa_circ_0001946 in IA patients and healthy people was significantly different (*p* < 0.05). Studies have shown that circ-CDR1as can specifically bind miR-7-5p through ceRNA mechanism, regulate downstream target genes expression, and participate in the pathological mechanisms of various cerebrovascular diseases (22, 41). Genes such as PARP1 and RAF1, which are important downstream targets of miR-7-5p, are closely related to various pathophysiological mechanisms. Dysregulation expression of these genes can affect the proliferation and migration of important functional cells such as microvascular endothelial and vascular smooth muscle cells, causing damage and weakness of the vessel wall, and promoting the development of atherosclerosis (42).

circRNA has good structural stability and can be released into extracellular space through exosomes, so it can be detected in body fluids such as saliva, milk, and plasma (43), these characteristics make the exploration of circRNA as a biological marker of disease widely concerned and deeply studied. In this study, we verified the results of high-throughput sequencing of circRNA simultaneously in two human specimens of cerebral artery tissue and peripheral blood. The analysis showed that certain circRNAs are differentially expressed in ruptured IA tissues and peripheral blood, there is a certain correlation between their expression patterns. For example, compared with the control group, hsa_circ_0007142 was down-regulated in IA tissues and peripheral blood, hsa_circ_0005571 and hsa_circ_0033144 were up-regulated in IA tissues, and down-regulated in IA peripheral blood. In the same patient, the expression pattern of hsa_circ_0007142 in tissue and peripheral blood were positively correlated, and the expression patterns of hsa_circ_0008433 and hsa_circ_0033144 in tissue and peripheral blood were negatively correlated. However, there was no significant difference in the expression of hsa_circ_0033144 and hsa_circ_0007142 in the follow-up population validation, which may be related to the influence factors such as the small number of tissue samples included in RNA-Seq, so the verification of further expansion of the sample size is needed.

However, these circRNAs with certain expression correlation in small samples of IA tissue and peripheral blood are still worthy of attention. This suggests that circRNA may have some correlation expression characteristics in central tissue and peripheral blood, which provides a theoretical possibility for the use of peripheral blood to diagnose IAs. In addition, we screened the three circular RNAs (has_circ_0008433, has_circ_0005571, has_circ_0001946) indicators closely related to IA rupture, and confirmed that they have certain reference significance for IA discrimination (AUC = 0.726, 95% CI: 0.668~0.784). This also indicated that circRNA may have potential values as a biomarker, and provides a new perspective for the future research of IA non-invasive diagnosis and treatment strategies. In the study, we also found that specific circRNAs also interact with individual factors such as age, thereby further confirming that IA is a complex disease caused by the combination of environment and genetic factors.

The study also has certain limitations. First of all, we used a structured questionnaire to collect the data of the research subjects in the case-control study, and tried to reflect the clinical status of the patient as objectively as possible, but there were still some deviations inevitably. For example, the information distortion caused by the subjective will or memory bias of the respondents may have a certain impact on the research results. In the follow-up research, the sample size can be further expanded, and cross-regional cooperation can be strengthened to precisely control confounding factors and make the results more scientific. Secondly, this study does not yet have the conditions for multi-center research, so there are certain deficiencies in sample representativeness. Multi-center research needs to be carried out in follow-up studies to diversify the target population, thereby improving the accuracy and accuracy of the research. Finally, due to the limitations of case-control studies, it is not yet possible to accurately determine the causal relationship between circRNA and IA rupture, so further functional tests are needed to verify.

## Data Availability Statement

The raw data supporting the conclusions of this article will be made available by the authors, without undue reservation.

## Ethics Statement

The studies involving human participants were reviewed and approved by the Ethics Committee of the Second Affiliated Hospital of Fujian Medical University (2018-50). The patients/participants provided their written informed consent to participate in this study.

## Author Contributions

SW, HL, and DK contributed to the study design. QinH, YS, QiuH, and YZ performed statistical analysis, interpretation, and drafted the manuscript. QiuH, YS, SL, YC, and XX contributed to data collection and laboratory test. QinH, YS, SW, and HL revised the manuscript. All authors contributed to critical revision of the final manuscript and approved the final version of the manuscript.

## Conflict of Interest

The authors declare that the research was conducted in the absence of any commercial or financial relationships that could be construed as a potential conflict of interest.

## Abbreviations

IA: intracranial aneurysm
circRNA: circular RNA
ceRNA: competitive endogenous RNA
CTA: computed tomography angiography
MRA: magnetic resonance angiography
DSA: digital subtraction angiography
OR: odds ratio
95%CI: 95% confidence interval
AUC: area under the ROC curve.
