# Supplementary material for: Association Between Circular RNAs and Intracranial Aneurysm Rupture Under the Synergistic Effect of Individual Environmental Factors
Source: Front Neurol. 2021 Mar 4;12:594835. doi: 10.3389/fneur.2021.594835 (PMC7969784; doi:10.3389/fneur.2021.594835)
Supplement: Supplementary file 1 [file Table_1.doc]

**TABLE S1. Analysis of baseline data of case and control group with IA rupture**

| **Variable** | **Case [n (%)]** | **Control [n (%)]** | **χ^2^** | **p** |
| --- | --- | --- | --- | --- |
| Gender  Male  Female | 196 (43.5) | 196 (43.5) | 0.000 | 1.000 |
|  | 151 (56.5) | 151 (56.5) |  |  |
| Age  ≤60  ＞60 | 298 (85.8) | 297 (85.5) | 2.621 | 0.105 |
|  | 49 (14.2) | 50 (14.5) |  |  |
| Marriage  Married  Other | 327 (94.2)  20 (5.8) | 329 (94.8)  18 (5.2) | 0.000 | 1.000 |

**TABLE S2. Univariate analysis of individual behavior factors**

| **Variable** | **Case Group [n (%)]** | **Control group [n (%)]** | **χ^2^** | **p** |
| --- | --- | --- | --- | --- |
| Education level |  |  | 64.424 | < 0.001 |
| Primary school | 143 (41.2) | 71 (20.5) |  |  |
| Middle school | 164 (47.3) | 156 (45.0) |  |  |
| University | 40 (11.5) | 120 (34.6) |  |  |
| Poison exposure  No  Organic reagents  Pesticides  Chemical materials | 238 (68.6)  87 (25.1)  10 (2.9)  12 (3.5) | 283 (81.6)  46 (13.3)  8 (2.3)  10 (2.9) | 16.930 | 0.001 |
| Sitting time |  |  | 8.578 | 0.003 |
| ＜6 h/day | 13 (3.7) | 32 (9.2) |  |  |
| ≥6 h/day | 334 (96.3) | 315 (90.8) |  |  |
| Sleeping time  ＜7 h/day  ≥7h/day | 323 (93.1)  24 (6.9) | 304 (87.6)  43 (12.4) | 5.964 | < 0.015 |
| Exercise  No  1~4 /week  ≥5 /week | 213 (61.4)  97 (28.0)  37 (10.7) | 134 (38.6)  160 (46.1)  53 (15.3) | 36.274 | < 0.001 |
| Tea drinking  No  1~4 /week  ≥5 /week | 186 (53.6)  121 (34.9)  40 (11.5) | 108 (31.1)  157 (45.2)  82 (23.6) | 39.815 | < 0.001 |
| Smoking  No  Quit smoking  Smoking now | 194 (55.9)  19 (5.5)  134 (38.6) | 229 (65.9)  38 (11.0)  80 (23.1) | 22.855 | < 0.001 |
| Drinking |  |  | 7.620 | 0.054*^a^* |
| No | 316 (91.1) | 302 (87.0) |  |  |
| 1~2 /week | 14 (4.0) | 31 (8.9) |  |  |
| 3~4 /week  ≥5 /week | 3 (0.9)  14 (4.0) | 4 (1.2)  10 (2.9) |  |  |
| Labour  Low intensity  Medium intensity  High strength | 209 (60.2)  92 (26.5)  46 (13.3) | 215 (62.0)  98 (28.2)  34 (9.8) | 2.074 | 0.351 |

Note: a is using Fisher's exact probability method

**TABLE S3. Univariate analysis of dietary habits**

| **Variable** | **Case [n (%)]** | **Control [n (%)]** | **χ^2^** | **p** |
| --- | --- | --- | --- | --- |
| Salty diet  No  Yes | 228 (65.7)  119 (34.3) | 284 (81.8)  63 (18.2) | 23.356 | < 0.001 |
| Fatty diet  No  Yes | 322 (92.8)  25 (7.2) | 319 (91.9)  28 (8.1) | 0.184 | 0.668 |
| Light diet  No  Yes | 187 (53.9)  160 (46.1) | 219 (63.1)  128 (36.9) | 6.078 | 0.014 |
| Pickled food |  |  | 2.157 | 0.142 |
| No | 279 (80.4) | 263 (75.8) |  |  |
| Yes | 68 (19.6) | 84 (24.2) |  |  |
| Cooking oil  Vegetable oil  Animal oil  Mixed | 235 (67.7)  36 (10.4)  76 (21.9) | 289 (83.3)  10 (2.9)  48 (13.8) | 26.583 | < 0.001 |

**TABLE S4. Univariate analysis of specific physiological indicators**

| **Variable** | **Case (n=347)** | **Control (n=347)** | **Z** | **p** |
| --- | --- | --- | --- | --- |
| Weight | 62.0 (55.4, 67.0) | 62.0 (54.0, 69.5) | -0.301 | 0.763 |
| Diastolic pressure | 86.0 (78.0, 97.5) | 76.0 (71.0, 81.5) | -9.842 | < 0.001 |
| Pulse pressure | 59.0 (46.0, 73.0) | 51.9 (46.0, 56.0) | -5.575 | < 0.001 |

**TABLE S5. Univariate analysis of specific biochemical indicators**

| **Variable** | **Case (n =347)** | **Control (n =347)** | **Z** | **p** |
| --- | --- | --- | --- | --- |
| Hb | 132.0 (120.0, 145.0) | 142.0 (131.0, 152.0) | -6.895 | < 0.001 |
| GLB | 26.6 (24.1, 29.9) | 26.7 (23.7, 28.8) | -1.226 | 0.220 |
| LDL | 2.5 (1.9, 3.2) | 3.0 (2.2, 3.5) | -5.493 | < 0.001 |
| TG | 1.0 (0.7, 1.4) | 1.18 (0.7, 1.6) | -2.767 | 0.006 |
| TCHO | 4.2 (3.4, 4.9) | 4.8 (4.1, 5.4) | -6.972 | < 0.001 |
| Ca^2+^ | 2.2 (2.1, 2.2) | 2.3 (2.2, 2.3) | -9.552 | < 0.001 |
| Apo-A1 | 1.3 (1.1, 1.4) | 1.4 (1.2, 1.6) | -6.683 | < 0.001 |
| Apo-B | 0.9 (0.7, 1.0) | 0.9 (0.8, 1.1) | -3.808 | < 0.001 |

**TABLE S6. Univariate analysis of disease history**

| **Variable** | **Case [n (%)]** | **Control [n (%)]** | **χ^2^** | **p** |
| --- | --- | --- | --- | --- |
| Hypertension  No  Yes | 203 (58.5)  144 (41.5) | 274 (79.0)  73 (21.0) | 33.799 | < 0.001 |
| Stroke  No  Yes | 319 (91.9)  28 (8.1) | 337 (97.1)  10 (2.9) | 9.020 | 0.003 |
| Diabetes  No  Yes | 336 (96.8)  11 (3.2) | 328 (94.5)  19 (5.5) | 2.230 | 0.135 |
| Family HBp  No  Yes | 203 (58.5)  144 (41.5) | 274 (79.0)  73 (21.0) | 33.799 | < 0.001 |
| Family Stroke  No  Yes | 319 (91.9)  28 (8.1) | 337 (97.1)  10 (2.9) | 9.020 | 0.003 |
| Family Diabetes  No  Yes | 327 (94.2)  20 (5.8) | 323 (93.1)  24 (6.9) | 0.388 | 0.533 |

**TABLE S7. IA-related circRNAs based on high-throughput sequencing**

| **circRNA ID** | **Expression** | **Host gene** | **Length(nt)** | **Log2 FC** | **p** |
| --- | --- | --- | --- | --- | --- |
| hsa_circ_0008433 | Up | PDE4B | 351 | 5.7026 | < 0.001 |
| hsa_circ_0033144 | Up | BCL11B | 369 | 3.8851 | < 0.001 |
| hsa_circ-_0005571 | Up | IFI30 | 658 | 3.6338 | < 0.001 |
| hsa_circ_0040809 | Up | BANP | 448 | 2.6193 | 0.002 |
| hsa_circ_0056285 | Up | RALB | 548 | 5.4045 | 0.008 |
| hsa_circ_0072309 | Down | LIFR | 580 | -4.5028 | < 0.001 |
| hsa_circ_0007142 | Down | DOCK1 | 427 | -4.2062 | 0.003 |

**TABLE S8. Analysis of general demographic characteristics of the research objects**

| **Variable** | **Case [n (%)]** | **Control [n (%)]** | **χ2/t** | **p** |
| --- | --- | --- | --- | --- |
| Gender(Male, %) | 69 (46.0) | 78 (51.7) | 0.963 | 0.326 |
| Age | 53.97±10.28 | 52.71±8.85 | -0.559 | 0.576 |
| Marriage (Married, %) | 136 (90.7) | 145 (96.0) | 3.485 | 0.062 |
| Culture (Middle school, %) | 82 (54.7) | 69 (45.7) | 3.158 | 0.076 |
| BMI (kg/m^2^) | 23.33±2.52 | 22.79±2.53 | -1.873 | 0.062 |

**TABLE S9. Diagnostic sensitivity and specificity of specific circRNA**

| **circRNA** | **SE** | **AUC (95% CI)** | **Sensitivity** | **Specificity** |
| --- | --- | --- | --- | --- |
| has_circ_0008433 | 0.030 | 0.703 (0.643~0.763) | 0.364 | 0.936 |
| has_circ_0005571 | 0.033 | 0.617 (0.552~0.683) | 0.393 | 0.800 |
| has_circ_0001946 | 0.032 | 0.658 (0.599~0.714) | 0.400 | 0.857 |
| Combined factors | 0.029 | 0.726 (0.668~0.784) | 0.793 | 0.564 |

**TABLE S10. ROC curve of IA related circRNAs and its combined diagnosis**

| **circRNA1** | **circRNA2** | **SE** | **Z** | **p** |
| --- | --- | --- | --- | --- |
| has_circ_0008433 | has_circ_0005571 | 0.034 | 2.56 | 0.011 |
| has_circ_0008433 | has_circ_0001946 | 0.041 | 1.09 | 0.274 |
| has_circ_0005571 | has_circ_0001946 | 0.043 | 0.96 | 0.337 |
| has_circ_0008433 | Combined factors | 0.017 | 1.38 | 0.169 |
| has_circ_0005571 | Combined factors | 0.033 | 3.32 | 0.001 |
| has_circ_0001946 | Combined factors | 0.034 | 2.01 | 0.044 |
